# Inhibition of Lyn kinase: A novel approach to treatment of Alzheimer’s disease

**DOI:** 10.1002/alz.087506

**Published:** 2025-01-09

**Authors:** Avi L Benitah, Timothy I. Richardson, Pathum M Weerawarna, Michael T Robo, Emily R Mason

**Affiliations:** ^1^ Indiana University School of Medicine, Indianapolis, IN USA; ^2^ Indiana Bioscience Research Institute, Indianapolis, IN USA

## Abstract

**Background:**

The TREAT‐AD centers aim to improve Alzheimer’s Disease (AD) research by offering free, high‐quality tools and technologies.

Lyn is a tyrosine kinase that belongs to the Src family kinases. The expression of Lyn and its activity have been implicated in AD. This class of proteins is involved in TREM2 mediated microglial activation and phagocytosis, a process which is beneficial for clearing neurotoxins such as Aβ oligomers in the brain. Lyn inhibition may activate microglia. Given the relationship between accumulation of Aβ and its exacerbation of neurodegenerative diseases such as AD, selective inhibition of Lyn has been proposed as a novel therapeutic approach to treating early‐onset AD. However, potent, selective, and brain penetrant Lyn inhibitors are unavailable to test this hypothesis.

**Method:**

We screened a variety of known kinase inhibitors to determine their activity towards inhibition of Lyn using the biochemical HotSpot kinase assay. With this data in hand, we identified imatinib as a starting point for the design of novel Lyn inhibitors. Structure‐based design and computational docking models were used to propose more active and selective Lyn inhibitors, which were synthesized. The activities were determined, and multiple parameter optimization (MPO) informed iterative Structure Activity Relationship (SAR) studies. The best compounds were evaluated in assays of microglia activation, and their drug metabolism and pharmacokinetic (DMPK) properties were determined.

**Result:**

A series of novel type II inhibitors are now available for testing. The results demonstrate a unique tail group provides the novel scaffold with potent activity and selectivity towards inhibition of Lyn, exceeding that of imatinib.

**Conclusion:**

Computational models, SAR, and MPO provided potent and selective Lyn inhibitors with good DMPK properties. Further studies are under way to determine the impact of these compounds on TREM2 mediated activation of microglia both in vitro and in vivo.

**Author List**:

Avi Benitah PhD,

Pathum M Weerawarna PhD,

Michael Robo, PhD,

Shaoyou Chu, PhD

Emily R Mason, PhD

Timothy I Richardson, PhD

